# Do health apps need endorsement? Challenges for giving advice about which health apps are safe and effective to use

**DOI:** 10.1177/2055207617701342

**Published:** 2017-04-10

**Authors:** Michelle van Velthoven, John Powell

**Affiliations:** Nuffield Department of Primary Care Health Sciences, University of Oxford, UK

Two decades ago, during the rapid emergence of the worldwide web as a popular source of health information, there was widespread anxiety among health practitioners, and especially the medical profession, that poor quality information on the internet could cause significant harm.^1^ Although there was never any consistent systematic evidence of harm beyond a few individual case reports,^2^ one response by many national health agencies was to call for, and in some cases develop, quality rating schemes.^3,4^ In 2000, an editorial in *The BMJ* by Tony Delamothe described these initiatives as ‘kitemarking the west wind’, referring to the difficulties in rating the quality of online health information and providing kitemarks for websites, and arguing that such schemes needed to demonstrate their benefit to population health before being pursued further.^5^

Just as the first generation of web technologies provided users with access to a vast quantity of online health information, now web2.0 interactivity and the rapid uptake of smartphones have led to an explosion in digital applications for health and wellbeing. An estimated 165,000 health apps were available in 2015 to the public, patients, health professionals and researchers.^6^ Health apps cover both ‘wellness’ apps, mostly for promoting healthy living (e.g. targeting physical activity, diet, sleep, stress), and ‘medical’ apps for diagnosing, self-managing, monitoring and treating conditions, providing decision-support and collecting health-related information.^7^

Regulation does exist for some health apps that are considered to be medical devices.^8^ In the United States, the Food and Drug Administration (FDA) launched a draft guidance on mobile medical apps in 2011, which was updated in 2015 and remains a work in progress.^9^ This guidance applies to health apps that meet the regulatory definition of a device, meaning those apps that are ‘intended to be used as an accessory to a regulated medical device, or transform a mobile platform into a regulated medical device’.^9^ In the United Kingdom (UK), the Medicines and Health Regulatory Agency (MHRA) published guidance in 2014 on health apps falling under their definition of medical devices and which should apply for CE marking (logo used in the European Union to confirm that a medical device is fit for purpose and adheres to safety legislation).^10^ While the exact number of health apps that have been registered as medical devices and FDA-cleared or received a CE mark is hard to determine, it is clear that so far only a very small proportion of health apps are regulated in this way (in 2013 there were approximately 100 FDA-cleared apps in total,^11^ and in 2016 only 24 devices connected to health apps and 12 medical devices were cleared).^12^ However, most health apps are not medical devices by these definitions, and are therefore currently unregulated by organisations such as the FDA and MHRA, and freely available to the public, regardless of their efficacy and safety.^13,14^

Just as with the concerns over the quality of health websites, health policy makers in different countries around the world are now considering whether wider endorsement of health apps is required, as a result of concerns about risks to patient safety and privacy related to existing apps.^8,15–21^ A recent review by the Commonwealth Fund showed that of 376 iOS apps and 569 Android apps for engaging patients with medical conditions, only 43% of iOS apps and 27% of Android apps appeared likely to be useful.^22^ Systematic assessments showed concerns about the clinical safety of several health apps.^19,21^ Concerns have also been expressed about selling data from health and wellness apps for commercial purposes.^23^

The European Commission,^15^ and national and regional governments, e.g. in the UK,^16^, Catalonia and Andalusia,^18,24^ are therefore currently developing guidelines for assessing the quality of health apps that include domains on usability, desirability, credibility, transparency, reliability, technical stability, privacy, safety and effectiveness. As with the multitude of instruments for rating health websites in 2000,^5^ we now have a considerable number of initiatives that rate the quality of health apps.^25^ But are these calls justified, and if such endorsement approaches were implemented would they bring net benefit to population health? Would (for example) a kitemarking process actually lead to increased recommendation and uptake of useful and safe apps, and reduced use of others? Or, as Delamothe argued about quality assessment of websites, should the advice be ‘don’t just do something, stand there’?^5^ Should people just decide for themselves which health apps to use? Most governments don’t see any need to endorse other sources of self-help such as books, and as described above there are existing regulatory approaches for apps which constitute medical devices. But do health and medical apps present a new challenge? The democratised, distributed nature of the internet means that a vast number of health apps of uncertain providence are produced and available to anyone with a smartphone. These may offer treatments that may or may not work, and potentially delay other forms of help-seeking. They may also collect confidential personal data and offer linkage to other health records or services. They are often offered to consumers in app stores where rankings are based on download statistics and user ratings, and previous work has shown that there is little correlation between user ratings and whether an app adheres to established evidence-based practices.^26^ Furthermore, when no criteria are set or guidance is given to app developers, the usefulness and safety of health apps might not improve, and there is little incentive for developers to thoroughly evaluate health apps or produce standardised meaningful information that can help people judge their safety and quality. Also, if an endorsement system required endorsed health apps to be registered, similarly for regulated medical devices, developers could be obliged to report adverse events related to their use.

There are challenges to an endorsement approach though. We do not know whether the public, or indeed health professionals, would use an endorsement system in choosing or recommending an app.^16^ Kitemarks may be ignored or misinterpreted, and those producing them take on some level of responsibility and liability. This links to the wider question of what does endorsement mean? Does it (for example) imply a recommendation for use, or a reassurance that the app in question meets minimum agreed standards? This is a challenge for policymakers in this area to address, and understanding how an advisory approach would influence health professional and consumer behaviour is a priority. No system could advise on or endorse all apps and many apps which can influence health behaviour (for example through diet or exercise) are not categorised as health or medical interventions. Understanding how to identify the apps to which an endorsement approach would add most value is another priority for health systems wishing to assess and provide advice on digital interventions. An alternative, which was adopted in the case of website quality, could be to publish codes of conduct.^5^ The European Commission has produced a voluntary Code of Conduct for data safety of health apps.^27^ A similar code could be developed for the quality of health apps, but rating quality is far more complex than adhering to privacy principles. For health apps that are not classified as medical devices, further specification of their risks could help identify health apps that are in most need of endorsement.^28^ There are challenges in identifying the level of scrutiny that a useful endorsement approach would require, and whether this extends to all dimensions of quality such as efficacy, safety, data privacy, usability and accessibility. Some previous efforts have been unsuccessful when their level of scrutiny was called in to question. In the US, the mobile app certification programme ‘Happtique’ was discontinued in 2013 because of concerns with data security apps the programme certified.^29^ Similarly in the UK, the National Health Services (NHS) health apps library for patient-focussed apps launched in 2013 was suspended in 2015. This followed studies questioning whether the listed apps had any evidence of efficacy,^30^ and demonstrating privacy issues with apps in the library.^20^ Any endorsement system needs to provide a level of assessment which meets the needs of its users, and to offer clarity as to what has been assessed.

In countries like the UK that apply Health Technology Assessment (HTA) criteria for health policy making, decisions to recommend health care interventions are mainly based on the safety, clinical effectiveness and cost-effectiveness of a health intervention.^31^ However, the current state of evidence for mobile health interventions, including health apps, is relatively immature as the clinical effectiveness of many health apps has not been established yet and cost-effectiveness information is mostly non-existent.^32–34^ Mobile health lacks tailored standards for evaluation, not surprisingly given its novelty.^35,36^ The interdisciplinary and innovative nature of mobile health is a strength but presents a challenge to traditional HTA approaches. For example, engineers and entrepreneurs might focus on rapid development cycles, whilst those from a clinical background concentrate on rigorous empirical evaluation of a stable intervention, which is often lengthy. Apps can be launched and offered direct to consumers with no endorsement, so what is the benefit to the developer? The trade-off between rigour and speed is a key challenge so as to provide a credible, trusted, rapid evaluation which does not stifle innovation.

In conclusion, health apps offer potential to improve health, but there are concerns about their safety, quality and usefulness. While various organisations are considering endorsement of health apps, there are many uncertainties about whether endorsement should take place and what consequences this will have. There are also uncertainties as to the level of scrutiny required by an endorsement process and the degree of trade-off between in-depth assessment and the promotion of innovation. Currently there is insufficient evidence on clinical effectiveness and cost-effectiveness for most health apps and standards for assessment and appraisal are in development. But providing no advice on health apps that are potentially insecure, unsafe or clinically not useful may lead to harm. Any endorsement process needs to demonstrate its benefit to health services or to the public’s health, by promoting the safe uptake of tools that bring health benefit or by reducing the uptake of apps which cause direct or indirect harm.
